# Psora

**Published:** 1898-07

**Authors:** J. M. Selfridge

**Affiliations:** Oakland, Cal.


### PSORA.

J. M. SELFRIDGE, M. D., OAKLAND, CAL.

Before taking up the study of anti-psoric remedies Hahnemann makes the following statement: "The *Psora*, a most ancient miasmatic disease, in propagating itself for thousands of years through several millions of human organisms, of which each one had its own peculiar constitution, and was exposed to very varied influences, was able to modify itself to such a degree as to cause that incredible variety of ailments which we see in the innumerable chronic patients with whom the external symptom (which acts vicariously for the internal malady)—*i. e.*, the more or less extensive eruption of itch, which has been driven away from the skin by a fatal art, or in whom it has disappeared of itself from the skin through some other violent incident."

From this and similar statements it has been the belief of some, and especially the allopaths, that Hahnemann's *Psoric* theory of chronic diseases is merely the common itch disease, which they have exploded, because the microscopist has discovered an insect—the *acarus scabei*—in the pustules. Having made this discovery, they think they have the laugh on Hahnemann and his followers, and it is their delight to hold them up to ridicule, as they did, not many weeks ago, when discussing the subject of homœpathic affiliation before the Regents of the University of California. But, had the Regents been more familiar with the teachings of Hahnemann, the laugh would have been at the ignorance of those who ridiculed Homœopathy.

Hahnemann says, "*Psora* is the *most ancient, most universal, most destructive,* and yet *most misapprehended* chronic miasmatic disease which for many thousands of years has disfigured and tortured mankind. . . . Psora is the oldest miasmatic disease known to us. . . . The oldest monuments of history which we possess show *Psora* even then in great development. Moses, three thousand four hundred years ago, pointed out several varieties. . . . The different names which were given by different nations to the more or less malignant varieties of leprosy (the external symptom of *Psora*), which in many ways deformed the external parts of the body, do not concern us and do not affect the matter," etc.

"The Occidental *Psora,* which during the Middle Ages had raged in Europe for several centuries under the form of malignant erysipelas (called St. Anthony's fire), reassumed the form of leprosy which was brought back by the return of of the Crusaders in the thirteenth century."

After speaking of the cleanliness which followed the introduction of linen underclothing, warm baths, and different diet, Hahnemann says, "The external horrors of the *Psora* within the space of several centuries were at least so far modified that at the end of the fifteenth century it appeared only in the form of the common eruption of the itch."

It will be well to observe that Hahnemann teaches that while *Psora,* under different names and forms, had troubled the human race long before historic times, it did not appear in the form of itch until about the end of the fifteenth century.

Owing to the repressive measures that have been used in the treatment of the itch, Hahnemann says the human race "is worse off from the change in the external form of the *Psora*—from leprosy down to the eruption of the itch." This is due to the fact that the itch is more easily repressed than the older forms of *Psora*, because when repressed or driven from the surface it becomes *latent*, and, as he says, "is the *most hydra-headed* of all the chronic diseases"—that is, when driven in by externally applied remedies, it shows itself in some other form, as phthisis, eczema, fibroma, etc. In fact it is the mother of all the chronic diseases the physician has to treat in this our day and generation, except the comparatively small number that are caused by latent syphilis and sycosis.

If we could eradicate *Psora* from every human organism the occupation of the physician would be well-nigh gone. This being true, it is evident that the first duty of the physician is to treat the *Psora* as manifested by the symptoms of a given case. To do this successfully a thorough knowledge of the antipsoric remedies is absolutely necessary. This Hahnemann recognized, and as soon as he became convinced of the truth of his theory he commenced the difficult and arduous task of proving remedies. What he accomplished he has left to us as an invaluable legacy, which is crystallized in the provings as found in his work on chronic diseases, which of itself is an enduring monument to his memory.

I have often wondered how he came to know the antipsoric value of different drugs. On this point he sheds some light when he says, "I have often been asked by what signs a substance may beforehand be recognized as antipsoric? But there can be no such external visible marks in them; nevertheless, while proving several powerful substances as to their pure effects on the healthy body, several of them by the complaints they caused showed me their extraordinary and manifest suitableness for homœopathic aid in the symptoms of clearly defined psoric diseases."

"Some traces of their qualities leading in this direction gave me in advance some hint as to their probable usefulness—*e. g.*, the efficacy of the herb *Lycopodium*, much praised in Poland for the *plica polonica*, pointed me to the use of the pollen," etc.

"The circumstance that some hemorrhages have been arrested by large doses of salt was another hint."

In a somewhat similar manner he obtained hints in regard to Guaiacum, Sarsaparilla, and Mezereum; while the earths, the alkalies, most of the acids, the neutral salts, and the metals were obtained from their pure symptoms while proving different drugs. This was continued until forty-eight antipsoric remedies had been thoroughly proven, and their curative powers verified.

In all cases "only those remedies have been acknowledged as antipsoric whose pure effects on the human health gave a clear indication of their homœopathic use in diseases manifestly psoric. Some of these medicines in their crude state seem to have a very imperfect, insignificant medicinal action (*e. g.*, common salt and the pollen of Lycopodium). Others (*e. g.*, Gold, Quartz, and Alumina) seem to have none at all, but all of them become highly curative by the preparation peculiar to Homœopathy." Why Hahnemann came to triturate Gold, Quartz, etc., he does not tell us. Possibly obtained the hint by using some of their salts.

At the same time, by this same peculiar process of preparation the most virulent poisons were made sufficiently "mild in their effects" to become curative in an almost incredible degree. This, as you know, is done by trituration and succussion on the centesimal scale. Again, by the process of trituration, insoluble substances, such as Gold, Silver, Platinum, etc., are rendered soluble in both water and alcohol, "a discovery invaluable to the healing art."

Another valuable discovery by Hahnemann is that when a potentized remedy is administered to a patient it is not neutralized by a substance that will antidote it in its crude form. For example, Natrum-carb., Ammonium-carb., Baryta, Lime. and Magnesia in a highly potentized form are not neutralized by taking vinegar after a dose of either of them has been taken. This is due, I think, to the fact that when a medicine is highly potentized its molecules become so active that they reach the diseased cells and do their perfect work before the crude substance has time to reach them.

In making his dilutions Hahnemann at one time recommended two powerful downward strokes of the arm, for the reason that a greater number increased the curative power of the drug in too great a degree. But, in his preface to antipsoric remedies (Tafel's translation, page 159), he takes this direction back in these words, "But during the last years, since I have been giving every dose of medicine in an incorruptible solution, divided over fifteen, twenty, or thirty days, and even more, no potentizing in an attenuating vial is found too strong, and I again use ten strokes with each. So I herewith take back what I wrote on this subject three years ago."

The experience of Hahnemann in the preparation and administration of his remedies led him, step by step, from the crude substance up to the thirtieth centesimal potency. Since his time experience has led some of his followers to the use of potencies unheard of or even unthought of by Hahnemann or the men of his time.

It has been found that the higher potencies will cure, oftentimes, when the lower will have no effect, or only aggravate the case.

It is customary, at the present time, to use a higher potency of the well selected remedy when a lower potency has been given without effecting a cure. But Hahnemann says, "if before he has used the thirtieth dilution he will now take one or two pellets of the twenty-fourth."

My own experience is that some of the most brilliant cures are made with the highest potencies. Such statements are frequently unpopular, and are often met with a smile of derision or a word of unbelief. But such expressions merely show that the person making them is not up-to-date in the art of prescribing. No person has the right to deny the truth of a statement or the curative virtues of the higher potencies until he has demonstrated by intelligent experiment that they are not reliable. I say intelligent experiment because careless prescribing is seldom or never followed by good results. If a remedy be indicated it may and frequently does cure in a low potency, provided some incompatible substance is not alternated with it. The single remedy and the smallest dose that will cure is the scientific art in medicine. In any case take Hahnemann's advice. I have found by experience that he is a reliable captain to follow.

